# Assessing the potential impact on health of the UK's future relationship agreement with the EU: analysis of the negotiating positions

**DOI:** 10.1017/S1744133120000171

**Published:** 2020-06-03

**Authors:** Nick Fahy, Tamara Hervey, Mark Dayan, Mark Flear, Mike Galsworthy, Scott Greer, Holly Jarman, Martin McKee

**Affiliations:** 1 Nuffield Department of Primary Care/Green Templeton College, University of Oxford, Oxford, UK; 2 School of Law, University of Sheffield, Sheffield, UK; 3 Nuffield Trust, London, UK; 4 School of Law, Queen's University Belfast, Belfast, UK; 5 Scientists for EU, UK; 6 School of Public Health, University of Michigan, Ann Arbor, USA; 7 London School of Hygiene and Tropical Medicine, London, UK

**Keywords:** Brexit, cross border healthcare, EU, NHS, UK

## Abstract

While policy attention is understandably diverted to COVID-19, the end of the UK's post-Brexit ‘transition period’ remains 31 December 2020. All forms of future EU−UK relationship are worse for health than EU membership, but analysis of the negotiating texts shows some forms are better than others. The likely outcomes involve major negative effects for NHS staffing, funding for health and social care, and capital financing for the NHS; and for UK global leadership and influence. We expect minor negative effects for cross border healthcare (except in Northern Ireland); research collaboration; and data sharing, such as the Early Warning and Response System for health threats. Despite political narratives, the legal texts show that the UK seeks *de facto* continuity in selected key areas for pharmaceuticals, medical devices, and equipment [including personal protective equipment (PPE)], especially clinical trials, pharmacovigilance, and batch-testing. The UK will be excluded from economies of scale of EU membership, e.g. joint procurement programmes as used recently for PPE. Above all, there is a major risk of reaching an agreement with significant adverse effects for health, without meaningful oversight by or input from the UK Parliament, or other health policy stakeholders.

## 1. Introduction

COVID-19 is likely to dominate the health policy agenda for most of 2020 and some time beyond. As a consequence, there is a risk that attention is diverted from other important issues, some having implications for the UK's ability to mount an effective response to the pandemic. The UK government faces the twin challenges of designing and implementing a response to one of the most severe COVID-19 situations in Europe while negotiating its future relationship(s) with the EU. How can it secure the ‘least worst’ outcomes for health and the NHS? The worst outcomes for health have been avoided so far, by the entry into force of the Withdrawal Agreement on 1 February 2020, a formal international treaty that establishes a transition period during which the UK is no longer an EU Member State but both parties agree to apply most EU law and policy as if it were. However, this period is scheduled to end on 31 December 2020, by which time the EU and UK should have concluded negotiations on their future relationship and agreed one or more legal texts. Despite the disruption caused by the spread of the virus and increasing voices calling for an extension to the transition period, the UK government has rejected asking for one and, as things currently stand, the UK's relationship with the EU will be on a ‘No Deal’ basis if an agreement is not in place by 31 December 2020.

While the EU−UK agreement(s) will primarily concern trade, with health a peripheral matter at best, trade agreements have important consequences for health (Gleeson and Labonte, 2019), and, as our previous analyses showed (Fahy *et al*., 2017, 2019) although all forms of Brexit are bad for health, some forms are worse than others.

This analysis considers the formal negotiating positions of the EU and the UK. The European Commission's negotiating mandate, annexed to the Council Decision 2020/226 of 28 February 2020, was agreed by the Council of the 27 Member States, having been debated by the European Parliament on 12 February 2020. The mandate formally binds the European Commission in its negotiations, subject to any further instructions from the Council. The EU is negotiating with the UK under its general ‘external relations’ powers, using a procedure which gives power both to the European Parliament and to domestic parliaments in the 27 Member States. The EU's position is that it is seeking ‘a new partnership agreement’ with the UK, which will be treated as a ‘third country’ post-transition. A draft EU text for the new partnership agreement was published by the European Commission's Task Force for Relations with the UK on 18 March 2020 (European Commission, 2020*b*).

The UK's negotiating position is not contained in legislation, has not been debated by legislatures in Westminster, Belfast, Cardiff or Edinburgh, and may, legally speaking, be changed by the UK government as it chooses. The UK's position is contained in a written ministerial statement given by Prime Minister Johnson to the UK Parliament on 3 February 2020, in Command Paper 211 published on 27 February 2020 (HM Government, 2020*d*), and in a draft legal text published on 19 May 2020 (HM Government, 2020*b*). The UK's position is that it is seeking a ‘comprehensive free trade agreement’ with the EU, along with a number of other supplementary agreements, some of which would be relevant for health in the UK.

The formal negotiations began in the first week of March 2020, but were temporarily halted in mid-March, when both chief negotiators, Michel Barnier and David Frost, tested positive for COVID-19. Negotiations restarted, using online communications, in mid-April 2020. This leaves a short window of opportunity during which the implications for health of any agreement should be understood by health policy stakeholders, and appropriate engagement with government (and the EU) should take place.

## 2. Method

As in our 2017 and 2019 papers, our method uses the WHO's health system building blocks to assess the likely effects on each aspect of the NHS in the UK. We have slightly modified this analytical frame to disaggregate a few topics which were previously considered together. This is necessary because of the way that implications of Brexit for health have unfolded. We focus on the two negotiating positions: the annex to EU Council Decision 2020/266 of 28 February 2020, and the UK Command Paper 211 of February 2020. Where the draft legal texts illuminate the negotiating positions, we have included those in our analysis.

We first examine and classify the compatibility of the negotiating positions: compatible, so agreement likely (green); basic aims compatible but differences mean agreement uncertain (yellow); no agreement likely because of fundamental incompatibility of aims (red) (Table 1, column 5). From that, we extrapolate a likely outcome for the agreement (Table 1, column 6), and then explain the likely short-term impact on health and the NHS (Table 1, column 7). We categorise these effects as unchanged (grey); positive (green); moderate negative (pink); major negative (red). To reach this conclusion, we are also comparing with our previous analysis of the Political Declaration (Fahy *et al*., 2019), also categorised as unchanged (grey); positive (green); moderate negative (pink); major negative (red) (Table 1, column 2), as against the comparative baseline of the UK remaining in the EU. As noted, we are working from the available political and legal texts, but of course the meanings and significance of those texts have not been tested.

**Table 1:** Summary of EU and UK negotiating positions and their implications for health Key: Column 2 Previous analysis of future relationship, based on political declaration: Grey - broadly unchanged; Green - positive; Pink - moderate negative; Red - major negative Column 5 Assessment of compatibility of aims: Green - agreement likely; Yellow - unclear; Red - no agreement likely Columns 7 Likely impact on health/NHS Grey - broadly unchanged; Green - positive; Yellow – too uncertain to analyse; Pink - moderate negative; Red - major negative

1. WHO Building blocks	2. Previous analysis	3. EU negotiating aims	4. UK negotiating aims	5. Compatibility	6. Likely outcome in agreement	7. Likely impact on health
**Workforce**						
Recruitment and retention of EU nationals in the NHS	No provisions facilitating recruitment and retention of NHS workers	Reciprocal rights and obligations in free movement	No provision for free movement of people	Relatively compatible	Restricted movement of EU workers	Increased difficulty in recruiting and retaining NHS and social care staff
Mutual recognition of qualifications	Weak ambition for MR of Quals	MR of Quals only ‘where in the Union's interest’	MR of Quals	Relatively compatible	Agreement in principle on MR of Quals	Broadly unchanged position
Employment rights for health workers	FTAs do not typically involve employment rights	Employment standards non-regression clause	Retain rights to modify employment standards	Level of standards likely to depend on wider agreement about trade and ‘level playing field’	Uncertain	Uncertain
**Financing**						
Reciprocal healthcare arrangements	Potential reciprocal health-care coordination	Reciprocal healthcare arrangements, no planned care abroad	Reciprocal healthcare arrangements, no planned care abroad (separate agreement)	Continued reciprocal healthcare arrangements	Continued reciprocal healthcare arrangements	Continued reciprocal healthcare arrangements, no planned care abroad
Capital financing for the NHS	Probably less capital financing than now	No provision	No provision	Compatible	No provision	Access to EIB stopped and access to capital financing reduced
Public spending		Free trade agreement likely to be associated with lower GDP, though less so than No Deal	Free trade agreement likely to be associated with lower GDP, though less so than No Deal	Broadly compatible		Significantly less public funding on a permanent basis than would otherwise be the case
**Medical products, vaccines and technology**						
Pharmaceuticals and other medical products	Potential for some weaker cooperation with EU on licensing and regulation of medicines than currently	FTA for goods; TBT to use international standards regulatory ‘floor’; no UK participation in regulatory structures	FTA for goods; TBT continued mutual recognition of potentially divergent standards; continuity in UK participation in technical structures	Degree of technical participation likely to depend on wider agreement about trade	Continued UK access to technical mechanisms if there is wider agreement on UK-EU trade	Increased costs of products associated with FTA
						Uncertain
Medical isotopes		Provision for continued supply from EU to UK	Provision for continued supply from EU to UK	Provision for continued supply from EU to UK	Continued provision	Continued access to medical isotopes
**Information**						
Comparable data between the UK and similar European health systems	No specific cooperation on health information	No provisions to retain UK data as part of EU data systems	No provisions to retain UK data as part of EU data systems	No provisions to retain UK data as part of EU data systems	No provisions	Cooperation within WHO; reduced comparability of data; loss of deeper cooperation on health information
Information exchange mechanisms on health-related aspects of free movement and substances of human origin	No specific cooperation on health information	Scope for cooperation where it is in the EU's interest	Mechanisms for some continued information exchange between the EU and the UK related especially to trade in goods	Degree of information sharing likely to depend on wider agreement about trade	Continued UK access to information exchange mechanisms for goods if there is wider agreement on UK-EU trade	Uncertain
Data protection	No specific cooperation on health information	Data protection based on EU law; adequacy decision envisaged	Independent UK data protection laws; UK seeks adequacy decision; UK recognises EU under adequacy decision	Separate autonomous data protection regimes	Separate and autonomous data protection regimes	Short-term compatibility but risk of long-term divergence with implications for cross-border services
**Service delivery**						
Working time legislation		Employment standards non-regression clause	Retain rights to modify employment standards	Level of standards likely to depend on wider agreement about trade and ‘level playing field’	Unclear	Uncertain
Cross-border care	Cross border health services not envisaged as part of future relationship (except in Ireland as part of peace process)	Continued UK participation in NI PEACE programmes	Provision for UK participation NI PEACE programmes	UK participation in NI PEACE programmes	No cross-border health services for Great Britain; continued in Northern Ireland	Continued UK participation in NI PEACE programme, but not other health-related EU programmes (eg: European Reference Networks)
**Leadership and governance**						
Public health	Scarce or no participation in ECDC	UK & EU autonomy to regulate public health	UK & EU autonomy to regulate public health	Agreement on autonomy on public health regulation	No substantive standards on public health in agreement	No short term change; scope for the UK to adjust public health regulation in the future
	Continued collaboration on public health at global level					
Competition and trade	NHS England no longer in perceived shadow of EU competition and public procurement law provisions	Dynamic alignment with EU competition rules; direct application of EU state aid rules; procurement based on GPA+	Transparency on harmful subsidies; no regulatory alignment, but commit to competition laws; public procurement excluded	Incompatible regarding alignment with EU competition rules. Public procurement rules aims compatible	Unclear; key point in negotiations	Uncertain
						Scope to relax public procurement rules for NHS England
	Reduced global influence over health in trade deals					Reduced global influence for UK over health in trade deals
Research	Continued participation in research envisaged but on worse terms for the UK; loss of global leadership and influence	Provision for UK participation in EU programmes as a third country	Provision for UK participation in research programme, but not health programme	UK participation in future research programme, but not other health-related programmes	UK participation as third country in some, but not all, health-related programmes	Continued UK participation in research programmes, but not other health-related programmes
Regulation of biomedical research*	Lack of shared regulatory framework impeding cross-border research	Services provisions envisage FTA-type approach, not regulatory alignment	No shared regulatory approach for services, but seeks de facto access for eg clinical trials	No common regulatory framework for cross-border research; technical cooperation depending on wider agreement about trade	Regulatory divergence; continued UK access to technical mechanisms if there is wider agreement on UK-EU trade	Uncertain
Scrutiny and stakeholder engagement	Volume of new legislation and executive powers under EU (Withdrawal) Act, plus new trade agreements, limits scrutiny and engagement					Risk of agreements with a substantive impact on health but which are not subject to normal processes of Parl scrutiny (or stakeholder involvement)

* not in original table

In this analysis, we are focusing *only* on the short-term effects in the months immediately post-transition (2021 into 2022), for health and social care in the UK. We have considered effects on health in the EU elsewhere (Hervey *et al*., 2021). We have included possible interactions of the effects of COVID-19 with the effects of the UK leaving the EU. Longer-term effects involve too many other considerations to allow a meaningful analysis of this type now, although we mention some possibilities. Neither do we consider the effects of a failure of the EU and UK to reach agreement during transition. Many of these would be the same as the ‘No Deal’ analysis in our 2019 paper, with appropriate adjustments for the renegotiated provisions for Northern Ireland, as well as for concurrent disruption to trade patterns and movement of people associated with COVID-19. However, it will be very difficult to distinguish economic disruption from a No Deal from that associated with COVID-19. We would also need to take into account the fact that the passage of time has meant greater preparedness for No Deal, on both the EU and the UK side. Many aspects of contingency planning relevant to health were put in place for the original ‘Brexit Days’ and these either remain in place or could be reactivated. Of course, all have cost implications.

## 3. Analysis and discussion

### 3.1 Health and social care workforce

The health workforce remains the most important challenge for the NHS post-Brexit. Although the formal position of EU nationals working in the NHS has not changed during transition, the move from ‘EU citizenship’ to ‘pre-settled’ and ‘settled status’, along with the ‘hostile environment’ that underpins the government's migration policy has had a chilling effect, and many EU nationals working within the NHS have already left, or are seeking to do so. Data from the Nursing and Midwifery Council show that an inflow of nurses and midwives from the EU into the UK until 2016 (which accounts for almost all the growth on the register from 2013 to 2016) has become a net outflow ever since (Nursing and Midwifery Council, 2019). Although there has been a significant increase in non-EU nationals, mainly from India and the Philippines, joining the UK nursing workforce, overall numbers joining the register having trained overseas remain below those in 2016 (Buchan *et al*., 2019). In November 2019, there were over 100,000 vacancies across NHS England (The Health Foundation, The King's Fund, and Nuffield Trust, 2018), of which almost 44,000 were in nursing, representing 12% of the nursing workforce, according to the Health Foundation (Buchan *et al*., 2019). The Coronavirus Act 2020 allowed for emergency measures to plug some gaps, for instance drawing on health professional students and recently retired staff. Notwithstanding this, the extent and nature of existing staffing gaps mean that the UK will be reliant on international health professionals for the foreseeable future, particularly in key areas, and especially in nursing. Visa extensions for some frontline health workers were announced in April 2020, but do not cover many of the lowest-paid health and social care workers across the UK.

The COVID-19 pandemic has reinforced the importance to health of the social care sector, which also faces growing staffing problems and has been especially reliant on EU migration. Data for England show that vacancy rates in the sector have steadily risen to 8%. There would be even more pressure without continued growth in the EU migrant workforce, which has risen rapidly to 115,000 (Skills for Care, 2019). The potential for a dramatic exacerbation of the situation if the end of free movement changes this has been widely commented on by researchers: the UK Government Migration Advisory Committee noted that with no action on wages in the sector ‘migrant workers will be necessary to continue delivering these services’ (Migration Advisory Committee, 2018, para. 5.31).

The EU and UK negotiating positions are consistent in that they reflect the ending of free movement of people between the UK and EU, and repatriation of immigration control to the UK. Both sides envisage limited ‘mobility’ provisions, visa-free travel for short-term stays, and arrangements for students, researchers and youth exchanges. COVID-19 measures are likely to mean reduced human migration between countries in general, although migration of essential staff, including NHS workers, will continue. The UK has offered concessions to international NHS staff already in the UK as part of its adopted COVID-19 response, including temporary changes to its ‘Tier 2’ immigration processes, which apply to NHS staffing. Obviously, outside the EU, the UK will not be able to benefit from cross-border collaborations to share health workers being developed to respond to disasters such as COVID-19.

Post-transition, the rules of the Common Travel Area (which allows free movement for British and Irish citizens between the UK and Ireland) will continue to apply. None of the proposed mobility provisions will replicate EU free movement law or the Withdrawal Agreement position. Recruitment of EU nationals to the NHS workforce will be on the same basis as recruitment of nationals from the rest of the world. The terms on which non-national NHS staff live and work in the UK under the new EU−UK relationship will be significantly less secure than those for EU nationals migrating within the EU, as they will be based only on UK domestic law. The lack of detail on this makes it difficult to estimate how many who come to the UK will choose to stay and work in the NHS following registration (Buchan *et al*., 2019).

The system planned for migrants from all other countries, European and non-European alike, was laid out in February 2020 in a UK Government policy statement (HM Government, 2020*e*). It uses a combination of shortage status, qualifications and salary to determine who can enter the UK for work. The system would mean that migrants would be allowed to take up almost any professional clinical role as long as that role is deemed to be in shortage, as they generally earn well in excess of the absolute threshold of £20,480 per year. Without shortage status, which is reviewed periodically and often subject to political intervention, some nurses and other clinicians outside London may not qualify.

Migration for the vast majority of social care roles would be impossible regardless of any shortages, as average pay is far below these levels, at around £16,200 per year in the predominantly independent English sector (Skills for Care, 2019). These positions also do not require the right level of qualifications. This has resulted in concern of exacerbating the already very difficult situation in the sector that was exposed by COVID-19.

These UK measures are understood to be responding to the desire of the British public, expressed in the EU referendum vote, to ‘take back control’ of domestic borders. However, the National Centre for Social Research (What UK Thinks, 2020) suggests that the UK population sees all health sector positions, no matter how lowly paid, such as ‘healthcare assistant’, as ‘skilled’; and that there is strong support (77% agree) for the new immigration system to prioritise people coming to work for the NHS. Sixty-seven percent of those polled also felt that ‘care worker’ is a ‘skilled’ job. These views have probably been strengthened by COVID-19. Nonetheless, unless the direction of travel of UK immigration law and policy changes to reflect this perception, given the likely outcome of the EU−UK negotiations on future relationship(s), it is likely to become more difficult for the UK to recruit and retain EU nationals in the NHS and social care workforce than it was pre-referendum. It is also worth noting the possible impact of COVID-19 on perceived safety of working in frontline care in the UK. If COVID-19 deaths of care and health workers are very high relative to other countries, this, in future could serve to dissuade foreign nationals from considering such work here.

In at least two respects, however, we can expect the future EU−UK relationship(s) to facilitate continued recruitment and retention of healthcare professionals. First, if agreement can be reached, employment rights such as working time (discussed below) may be retained. Second, both the EU (Council of the European Union, 2020, para. 43) and the UK (HM Government, 2020*d*, para. 48–9) seek a framework for the mutual recognition of qualifications, although the EU's position is that this is only where it is in the EU's interest. Although the overall context in which this part of the EU−UK Agreement will be found (services and investment) is likely to be modelled on a much less close trade relationship than EU membership (such as EU−Canada, or EU−Japan), the key difference in the context of professional qualifications is that the EU and the UK are *beginning from* common frameworks and current mutual recognition, rather than *building towards* such a position. Although the new rules will not be enforceable by individual health professionals, as under EU law, in practice individual enforcement is the exception. The coordination of qualification rules through collaborative processes is more important. Both the EU's [European Commission, 2020*b*, para. SERVIN 1.14(2)] and the UK's (HM Government, 2020*a*, Articles 8.4 & 13.13) draft texts envisage a new institutional framework for such coordination: a Specialised Committee on Trade/Trade in Services and Investment/Sub-Committee on Recognition of Qualifications. In practice, this may result in a relatively unchanged regulatory environment, unless the UK decides to depart significantly from medical professional qualifications standards set by the EU. While most relevant professional organisations support alignment, some, such as the Royal College of Surgeons, suggest some changes to domestic law would be desirable.

What will certainly change is the easy exchange of information between the EU and UK about professionals providing medical services across borders where there are concerns about fitness to practise, using the Internal Market Information (IMI) system. Running since 2008, the IMI is a multilingual online tool through which national public authorities of the EU Member States exchange information about the functioning of the internal market, including to improve professional mobility. By 2018, over 4000 alerts had been sent concerning disciplinary sanctions taken against doctors, just over 30% of all alerts sent (European Commission staff working document, 2018). The UK has been one of the most frequent users of the system, reflecting the number of doctors it employs from EU Member States. Although the IMI Regulation envisages the possibility of access for third countries, this is under stringent conditions, including the application of EU law in the relevant third country. No non-EEA country (not even Switzerland) currently has IMI access. Unless the EU changes its position, exchange of data about medical professionals' fitness to practise will no longer be possible through IMI after the end of transition. This will mean that, unless another agreement covers such information exchange, the UK will need to increase vigilance and oversight of fitness to practise in order to maintain standards of patient care at pre-transition levels.

### 3.2 Financing

#### 3.2.1 Reciprocal healthcare

In previous articles we highlighted the potential loss of reciprocal healthcare for people travelling between the EU and the UK, and UK nationals who have retired to EU countries (Fahy *et al*., 2017). Fortunately, agreement on some form of continued reciprocal healthcare arrangements is a specific objective of both sides (HM Government, 2020*d*, para. 17–18; Council of the European Union, 2020, para. 58), though this is one of the topics where the UK is seeking a separate side agreement rather than being part of the single integrated agreement sought by the EU, creating the risk of dispute over form even if there is agreement on the substance.

The EU's draft legal text includes detailed provisions in its draft Protocol on Social Security Coordination, which largely mirror existing EU law in Regulation 883/2004, but with one notable omission. While this draft text retains provisions to receive immediately necessary care while travelling between the UK and the EU and would ensure continued healthcare coverage for UK nationals who have retired to EU countries, it omits provisions for travel between the UK and the EU for the purpose of receiving healthcare. This is also the case for the UK's draft Agreement on Social Security Coordination (HM Government, 2020*c*).

Agreement is likely to be reached on this topic, as the negotiating mandates are well aligned (and have been detached from wider discussions around free movement of persons, which is the basis for the regulations in EU law), and this will avoid most of the negative outcomes that we identified in previous analysis. However, removal of provisions facilitating travel for the purpose of receiving care elsewhere takes away the ability of UK patients to seek care in a specialist centre elsewhere in Europe, for example, or for UK pensioners now resident in Spain to return to the UK to seek healthcare near to their families.

#### 3.2.2 NHS financing

The EU has been a significant source of funds for infrastructure investment by the NHS, with the European Investment Bank having provided it with over £3.3 billion in low-cost capital. Neither negotiating mandate envisages continued UK participation in such EU financing structures, which will reduce access to financing for infrastructure improvement. However, as we described in our previous analysis, the greater impact on NHS finances is likely to come from the wider long-term negative economic impact of leaving the EU. The COVID-19 pandemic changes the wider economic context for the NHS and the economy as a whole. Quite how much impact leaving the EU has on the economy in the short term will be bound up with the much wider impact of the pandemic. The Government has mobilised sums of money to tackle the pandemic that would have been unthinkable just a short while ago, but it is not yet possible to assess what these will mean for the NHS, the wider economy, or the long-term public finances.

However, the negative impact on GDP of shifting the UK's trading with the EU to a free trade agreement will be permanent, as will its impact on funds available for the NHS and social care. The Treasury's Long-term economic analysis of EU exit in 2018 calculated that a typical free trade agreement Brexit would result in UK GDP being between 4.9% and 6.7% less than it otherwise would be after 15 years (HM Government, 2018). Though this is an uncertain area for forecasting, the vast majority of studies similarly predict that the UK economy will be smaller, and many suggest an even larger impact (Tetlow and Stojanovic, 2018). The UK in a Changing Europe has concluded that any such reductions in projected GDP will feed directly into public spending, after the end of contributions to the EU is taken into account (The UK in a Changing Europe, 2018).

As an illustration, taking current GDP of £2.2 trillion per year, the UK in a Changing Europe methodology and the more optimistic Treasury estimates would imply a reduction of over £30 billion in annually available public spending. This would limit the ability of any government to increase funding for the NHS, cover more people against the costs of social care, and invest more in preventing disease.

### 3.3 Medical products and substances of human origin

The EU−UK agreement will cover trade in products, including pharmaceuticals, medical devices, medical equipment [including personal protective equipment (PPE)], and consumables. Substances of human origin (blood, plasma, human tissue and cells, human organs) are not conceptualised as products in EU law, and are not explicitly mentioned in the negotiating positions. Products made from substances of human origin will be included in the terms of the agreement. As the main focus of the envisaged agreement is trade in products, this aspect of post-transition will have the least effect on health and the NHS. One area that is likely to be secured is continued access to medical isotopes [HM Government, 2020*d*, para. 25(d); Council of the European Union 2020, para. 85]. The expected outcomes are far from the potentially life-threatening effects on supply chains associated with No Deal, but that is not to say that there will be no effects. Trade in relevant products, and the components which are used to make those products, will continue post-transition on the basis of an agreement which goes some way – but not all the way – to replicating the trade patterns that apply before the end of transition. The UK's negotiating position seeks to go further in this regard than the EU's.

Both parties seek to secure a free trade area in goods: in other words, there will be no tariffs or quotas restricting trade. But there will be a number of other hindrances, and these will impede trade in the products that the NHS uses as much as any other sector. Hindrances include rules of origin, possible anti-dumping and anti-subsidy duties, and economic safeguards, to protect domestic producers. Although the agreement is likely to include ‘customs facilitation’ (measures to reduce ‘red tape’ at borders), there is no avoiding the need for significant changes to the (electronic) paperwork needed for products, and components, coming from the EU into the UK, and time taken to process them. The costs of these new processes are likely to be passed on to consumers of the corresponding products, which in this case is the NHS. Both independent research and the Government's own modelling suggest a free trade agreement-type relationship with the EU would increase costs of medicines, devices and equipment to the NHS by around 5% – not as much as No Deal Brexit (7.5%), but a significant cost to the NHS budget, estimated in November 2018 (Health and Social Care Committee, 2018) at £400 million per annum.

Further, the EU−UK agreement will address ‘non-tariff barriers’ (measures other than direct tariffs that have the practical effect of restricting imports or exports of goods or services). Here, the UK and EU positions diverge to some extent, which makes it difficult to assess likely outcomes and therefore implications for health and the NHS. One of the EU's most impressive achievements is the removal of these de facto hindrances to free trade, with consequent benefits arising from economies of scale. In part, the EU has done this through deep cooperation in setting safety standards, using European agencies, made up of experts from its Member States, which agree standards that all EU countries can support. This system operates for medicines licensing, and links to clinical trials regulation. The EU also has the CE system of automatic recognition of the assessments of private standards entities that check conformity with technical regulations, irrespective of where those bodies are established in the EU. This system operates for medical devices and equipment. Compliance with these dense EU rules, and submission to oversight and dispute settlement through EU or EEA institutions, secures access to the EU's market.

The UK's negotiating position suggests that the UK seeks access to that market on terms better than those, say, for Canada, but without consenting to the package of internal market law that the EU insists is indivisible. In many respects, the UK's negotiating documents are directly equivalent to the EU−Canada Comprehensive Economic and Trade Agreement (CETA). But in a few, the UK wants to go further, and some of these involve medicines, devices and medical equipment (including PPE). For example, unless covered in the EU−UK Agreement, UK-based standards entities will no longer be recognised by the EU post-transition. As such, for instance, firms marketing medical devices in the EU will need a CE mark from an EU-based body. Further, under the new Medical Devices Regulations, firms will also have to have an EU-based authorised representative who will be liable for defective devices along with the manufacturer, thus ensuring legal redress by patients within the EU. The EU's position refers only to cooperation with international standards bodies, whereas the UK proposes detailed provisions on mutual acceptance of the results of each other's conformity assessments, including UK access to the EU's electronic information exchange systems for these administrative purposes [HM Government, 2020*a*, Annex 5A, Article 5(1)]. The UK's position is, in effect, to seek continuity post December 2020, by proposing an automatic continuity of recognition of UK standard setting bodies' ability to recognise compliance with EU standards.

Another example is the UK's proposed annex on medicinal products (HM Government, 2020*a*), for which there is no equivalent in the EU text. This annex aims to ‘reinforce competitive market conditions based on principles of openness’ and *‘maintain* cooperation to foster *continued* mutually beneficial development in trade’ (italics added), suggesting a continuity that is absent in the EU position. Alongside good manufacturing practice recognition (which is covered by trade agreements like CETA), mutual recognition of pharmaceutical batch release control regulations (based on the EU−Israel agreement on conformity assessment (*Protocol to the Euro-Mediterranean Agreement Establishing an Association between the European Communities and Their Member States, of the One Part, and the State of Israel, of the Other Part, on Conformity Assessment and Acceptance of Industrial Products,* 2013) which covers some, but not all, pharmaceuticals, and which involves Israel aligning with EU rules), the UK position seeks to include mutual recognition of good clinical practice. Many UK-based companies supplying the health sector have in fact already set up subsidiaries in the EU, so that they can ensure the compliance with EU standards and procedures, such as batch-testing of medicines, necessary to continue to supply the larger EU market: the UK position of mutual recognition would make it easier for UK-based companies to continue to supply that market. Furthermore, though exactly how access would be arranged is left for subsequent technical discussions, the UK seeks continued sharing of information about adverse post-market effects of new medicines (pharmacovigilance) and clinical trials information, currently done through the European Medicines Agency, again neither of which is covered by CETA.

By contrast, the EU does not seek agency-level arrangements in areas relevant to health and the NHS, although the future EU−UK relationship is likely to continue some agency-level cooperation (for instance in aviation, and nuclear energy). While in the EU, the UK was part of a single licensing system for medicines marketed anywhere in the EU, based on a single clinical trial system, applicable to all EU cross-border clinical trials. The EU's position is that the UK, by deciding to leave the internal market and eschew formal regulatory alignment with its rules, has taken itself outside the clinical trials system processes, and their benefits. These benefits include the ability to access larger patient populations (important for trials of medicines for rare diseases); to sell to a large market without the costs of altering technical standards; and to respond to pharmacovigilance information drawn from a large patient population. From the point of view of the EU, the UK will not be able to be part of the processes overseen by the European Medicines Agency, which relocated from London to Amsterdam on 1 March 2019, or the EU's technical standards processes for medical devices or equipment. Given the divergence of the positions, the agreement is likely to require only that the UK and the EU work through international standards bodies, such as the International Organization for Standardization (ISO) and the International Council on Harmonisation (ICH) (Flear, 2018).

That means, though, that the EU and the UK will each be permitted to require that products sold in their markets meet their own safety standards. The Medicines and Medical Devices Bill HC Bill (2019–21) [90], when it comes into force, will allow ministers to amend domestic law on medicines and medical devices without primary legislation. The delayed conclusions of the Cumberlege Review (Independent Medicines and Medical Devices Safety Review, 2020) on safety of medicines and devices are likely to feed into decisions in this policy space. The Bill envisages that the relevant executive authorities may amend all relevant areas of law currently regulated at EU level, subject to a duty to pay attention to safety and availability of medicines and devices in the UK, as well as ‘the attractiveness of … the UK as a place in which to conduct clinical trials or supply human medicines’. The Bill has been welcomed by some industry actors, who see the ability to avoid primary legislation as enabling the UK regulatory environment to respond rapidly to technological change. Others are concerned about the costs of regulatory drift from the EU, and especially about the lack of a time-limit on executive power in such broad policy areas. Compliance with obligations in the EU−UK agreement will provide some constraints on the UK's and the EU's ability to require regulatory standards higher than those that currently apply without scientific justification, as to do so would introduce ‘non-tariff barriers’, contrary to the agreement. But these obligations are mainly procedural: each party to the agreement will retain power to determine its own safety and health standards. Because the UK will be outside of the EU's medicines licensing, clinical trials approvals and medical device standards decision-making processes, it will be unable to influence these directly, except through international bodies like the ICH. There will be no constraints on the UK adopting *lower* standards than the EU's, other than soft incentives to align with the larger EU market, and the vague objective of convergence based on international standards. Non-alignment is likely to mean increased costs in terms of regulatory compliance (as companies would have to show compliance with two sets of processes and standards), and less UK bargaining power with the global industry. Those factors could mean that UK patients faced delays in access to innovative medicines and vaccines.

Finally, the EU has used joint procurement processes, for instance for PPE, ventilators, and laboratory equipment to respond to COVID-19 (European Commission, 2020*c*), as the European Commission sought to do with swine flu vaccines in 2009, using its bargaining power as a bloc. The UK belatedly indicated it would join that joint procurement for COVID-19 in late March 2020: it will be unable to join similar initiatives post-transition. Also, after December 2020, the UK would be subject to any export bans the EU imposes on essential products. This may affect the UK's ability to source medical equipment, medicines or vaccines necessary to respond to a second wave of COVID-19 after that date. During the first wave the EU banned the export without authorisation of face shields, masks and many other types of protective equipment for a period of six weeks (European Commission, 2020*a*).

### 3.4 Information

Three information dimensions of the EU−UK agreement are information exchange within the EU's internal market (discussed above); comparable data about health and health systems; and regulation of data sharing, in particular protection of personal data.

One of the advantages of European cooperation is comparing and learning across countries, and the EU's comparative data is a key resource for doing so. The European statistical system coordinated by EUROSTAT, the EU's statistical office, is central, but neither the UK nor the EU seeks for the UK to remain integrated within this system, which is likely to lead to divergence of data over time and reduced comparability. Other specific mechanisms for collecting and sharing comparable data include the European Centre for Disease Prevention and Control (the ECDC). Data on COVID-19 illustrate the impact of this. While some data are available through a variety of global sources, the ECDC is able to provide enhanced data through more in-depth collection and reporting for the EU (and the UK, during the transition period). While the UK will remain part of the World Health Organisation (WHO) and thus remain integrated within the WHO's data structures, these inevitably do not have the same degree of coverage or harmonisation as EU data systems. The EU's draft legal agreement does also include the possibility of the EU granting temporary access to its Early Warning and Response System (EWRS), which is a system for sharing information and coordinating response between governments to public health threats such as COVID-19, though it is not included in the UK's negotiating position.

Both negotiating mandates envisage data protection rules being established autonomously by the EU and UK, with each then allowing flows of personal data to the other provided that the data protection rules of the other side are deemed to provide adequate protection (HM Government, 2020*d*, para. 59; Council of the European Union, 2020, para. 13). There will no longer be a common mechanism between the UK and the EU for the protection of personal data, nor any ambition to establish one. In the short term, this is unlikely to cause serious issues, but in the longer term some divergence seems likely, with implications for cross-border trade in services requiring the processing of personal data between the UK and the EU.

### 3.5 Service delivery

#### 3.5.1 Working time legislation

The EU has emphasised the importance it attaches to a ‘level playing field’, which addresses health and safety standards, including legislation on working time. The EU's negotiating mandate seeks a commitment that the UK's labour standards will not be reduced below the level currently required by EU law (Council of the European Union, 2020, para. 101), and will be effectively enforced – which would mean that provisions on limiting working time could not be weakened in the future, either in law or in practice. The UK's negotiating position does not provide for a similar non-regression provision. Rather, it envisages labour law being developed autonomously by both sides, with only a more general commitment not to reduce labour laws and standards ‘in order to encourage trade or investment’ (HM Government, 2020*d*, para. 75).

These aims diverge, with the EU seeking guarantees that the UK will maintain labour law protections that currently exist, and the UK seeking the right to change them. What agreement is reached will depend on the wider result of negotiations regarding the ‘level playing field’ sought by the EU but rejected by the UK. If there is no agreement here, this may compound the effect of more restrictive immigration rules by making employment conditions in the NHS and social care less attractive than those elsewhere in the EU, making it harder to recruit staff from the EU than otherwise. This may be particularly challenging for staffing in Northern Ireland. Irrespective of agreement, UK domestic law or policy may *de facto* embed aspects of a level playing field: for example, junior doctors in England have effectively secured continued application of the EU's working time rules via their contracts.

In the short term there is unlikely to be any immediate impact, as there are unlikely to be immediate changes to labour law even if there is agreement on the UK's scope to do so, or No Deal. In the longer term, it seems likely that any agreement acceptable to the UK would include scope to make longer-term changes to working time legislation, so the issue of what is appropriate working time for junior doctors is likely to return.

#### 3.5.2 Cross-border care

The situation with cross-border care is quite different for Northern Ireland and Great Britain. Both negotiating mandates envisage continued UK participation in the cross-border PEACE programmes between Northern Ireland and Ireland (a strand of the EU's structural funds specifically promoting cross-border cooperation between Northern Ireland and the border counties of Ireland), including their health elements. However, the more general provisions on seeking, or providing, cross-border care (for rare diseases, for example) are not included in either negotiating mandate. This includes the reciprocal healthcare arrangements described above, as well as UK participation in cooperation frameworks such as the European Reference Networks. There are also no specific provisions on the cross-border provision of services such as telemedicine, or ‘back office’ services such as tele-radiology. The alignment of both sets of negotiating mandates suggests that agreement is likely to be reached on this approach, meaning that cross-border healthcare cooperation will continue for Northern Ireland but not for the rest of the UK.

### 3.6 Leadership and governance

#### 3.6.1 Global public health standards

Both the EU and the UK negotiating positions conceptualise public health matters as concerned with food safety, animal, and plant health, as covered by the World Trade Organization (WTO)'s Sanitary and Phytosanitary Agreement. The EU−UK trade agreement will prevent use of such standards to protect domestic markets, and require that both the UK and the EU base such standards on scientific assessments, though a similar obligation has not prevented disputes in the WTO context, for example about genetically modified food. The EU and the UK will undertake to cooperate in relevant international fora, including through sharing information. This arrangement will reduce the ability of health stakeholders to hold the UK government to account, because these fora are less transparent than the EU, and because many of them, e.g. WTO, do not prioritise health. Nothing in the EU−UK agreement will require the UK to align with the EU's standards, leaving the UK scope to adjust its public health regulation in the future. The EU seeks control over safety standards for imported food. By contrast, the UK seeks equivalence recognition along the lines of CETA or the EU's draft text for an agreement with New Zealand on veterinary standards (The Council of the European Union, 1997), which would reduce border controls and certification. In this area there is a major complication as it is likely to be difficult to reconcile US demands for a future trade deal with the UK with those by the EU.

There is no provision for cooperation on broader public health matters, such as tobacco regulation or communicable disease control. The implication is that continued cooperation will take place only within the framework of the WHO and other specialised agencies, where the UK's soft power as a relatively small state is likely to be diminished in comparison with its power as part of a common EU voice.

#### 3.6.2 Competition and trade

The UK and EU negotiating mandates are not compatible in terms of competition or state aids rules. The UK seeks commitments only to transparency in state aids, and to maintain competition laws. The EU seeks dynamic alignment (i.e. commitment to track EU standards) on a range of ‘level playing field’ commitments, including state aids and competition law. The lack of compatibility means outcomes are uncertain, and effects on health and the NHS consequently impossible to predict. On public procurement, the UK does not want to go beyond the minimal provisions of the WTO's Agreement on Government Procurement. This would mean that NHS England would no longer operate in the shadow of EU procurement requirements, which are felt to drive inefficient conduct in purchasing practices. However, outside the EU's trade structures, the UK's scope for influencing the place of health in trade deals is diminished.

#### 3.6.3 Participation in research programmes

Both negotiating mandates make provision for continued UK participation in EU-funded programmes as a third country, although the UK is only seeking participation in a relatively narrow set of EU programmes. This includes the research programme and the PEACE programmes in Northern Ireland, but does not include other health-related programmes, such as those financing European Reference Networks. The negotiations therefore seem likely to enable continued participation by UK research entities in wider European research programmes and the PEACE programmes in Northern Ireland, but not other activities with a more direct focus on health.

#### 3.6.4 Regulation of research

Participation in EU research programmes is only one aspect of the effects of the EU−UK agreement on biomedical research, and consequent effects on health and the NHS. The regulation of clinical trials is also important. This is complex, because it covers both cross-border trade in services (the service of the research itself); trade in goods; and data sharing. Health-related services are not explicitly covered in either negotiating text: despite the reality of many clinical trials, they are not conceptualised as a cross-border service, like transport, in these negotiations. As explained above, despite the political rhetoric of the Johnson government, the detail of the legal texts suggests that the UK seeks continuity in mutual recognition of what will be separate regulatory standards for ‘good clinical practice’, required to secure market access for medicines. The UK's position also implies ongoing data sharing using the EMA's systems, whereas the EU seeks a clean break in terms of such ‘internal market’ access. The divergence of positions here makes it very difficult to determine likely outcomes. The EU's position (and No Deal) would mean UK and EU clinical trials approval processes would have to be run separately. This raises concerns that the consequent increases in administrative burdens, especially if divergence arises either through deliberate regulatory choices, or just because of lack of practical, granular cooperation, will lead to reduced collaborative EU−UK research activity. The consequence would be fewer trials and a reduction in patient access to new treatments in both the EU and the UK.

## 4. Conclusion

Health is not a central issue in these negotiations, but their outcome will still have a significant impact on health. The largest direct impact on health and social care is likely to be on workforce, with no provisions to facilitate free movement for those working in health or social care (and even an agreement on mutual recognition of qualifications may not be that helpful in practice). One major negative direct impact seems likely to be avoided, with agreement likely on continued reciprocal healthcare arrangements avoiding UK citizens living in the EU being left at risk of lacking healthcare, though facilities have been removed that enabled patients to seek healthcare in the EU when needed. Direct disruption to health and social care in Northern Ireland also seems likely to be minimised through continued participation in the PEACE programmes.

The largest impact overall is likely to come through the predicted negative impact on the future economic growth of the UK, as described above, with those reductions in growth feeding through into reduced capacity to fund health and social care alongside other public services. More broadly, there is likely to be a progressive divergence of regulatory regimes related to health, with potential longer-term impacts in areas such as availability of medicines and participation in biomedical research. In particular concerning health-related products, the impact of the outcome of the negotiations will turn on what overall agreement is reached (if any) that resolves the tension between the UK's desired *de facto* access to the EU's internal market and the EU's aims for a ‘level playing field’.

The lack of health as a cross-cutting objective for these negotiations is disappointing, and in the case of the EU is arguably a breach of its Treaty obligations to integrate health in all policies (Article 9, Treaty on the Functioning of the European Union). From the UK perspective, as any agreement reached with the EU will be the reference point for future UK trade agreements (including with the United States), there is an opportunity to defuse tensions around standards and threats to the NHS by including health as a regulatory ‘floor’ in these negotiations. This could be achieved by including health-related protections as part of the ‘level playing field’ provisions and including a commitment to maintaining at least the current levels of health protection within the UK.

This assessment and avoiding negative impacts such as on cross-border care is provisional, and the risk of No Deal remains. The likely immediate impact of No Deal on supply chains and availability of key products is smaller than it would have been without the current transitional period, as both governments and other actors have had time to put in contingency plans. However, risks still exist; those plans are largely not publicly available and thus cannot be fully scrutinised, and the COVID-19 pandemic has put all aspects of health and care systems and wider society under immense strain, so there is even less slack to accommodate problems in the event of No Deal.

Many issues are raised for health and care by the UK leaving the EU which go beyond the scope of these negotiations. A new strategy is needed for ensuring appropriate health and care staffing with much reduced migration from the EU. A new approach to regulation of pharmaceuticals and other health-related products may also be needed, if the UK positions itself outside one of the two principal global regulatory jurisdictions.

Finally, the continued lack of transparency around the negotiations and lack of involvement of stakeholders raises serious concerns. The COVID-19 pandemic has underlined the need for central government to be transparent to enable effective analysis and input from stakeholders, especially in a technical area such as health. For these negotiations and for the series of trade negotiations that will follow in the years to come, it is vital to establish transparent processes that enable scrutiny and broad-based stakeholder input throughout.
